# Detection of multiple drug-resistant *Trypanosoma congolense* populations in village cattle of south-east Mali

**DOI:** 10.1186/1756-3305-5-155

**Published:** 2012-08-01

**Authors:** Erick O Mungube, Hervé S Vitouley, Emmanuel Allegye-Cudjoe, Oumar Diall, Zakaria Boucoum, Boucader Diarra, Yousouf Sanogo, Thomas Randolph, Burkhard Bauer, Karl-Hans Zessin, Peter-Henning Clausen

**Affiliations:** 1 Institute for Parasitology and Tropical Veterinary Medicine, Freie Universität Berlin, Königsweg 67, D-14163, Berlin, Germany; 2 Kenya Agricultural Research Institute, Katumani Research Centre, P.O. Box 340–90100, Machakos, Kenya; 3 Centre International de Recherche-Développement sur l’Elevage en Zone subhumide (CIRDES), 01BP 454, Bobo-Dioulasso 01, Burkina Faso; 4 Central Veterinary Laboratory, Pong-Tamale, P.O. Box TL 97, Tamale, Ghana; 5 FAO Regional Office, P.O. Box GP 1628, Accra, Ghana; 6 Laboratoire Central Vétérinaire (LCV), BP 2295, Bamako, Mali; 7 Pan African Tsetse and Typanosomosis Eradication Programme (PATTEC) Mali, BP 9125, Bamako, Mali; 8 International Livestock Research Institute (ILRI), P.O. Box 30709, Nairobi, 00100, Kenya; 9 International Animal Health, Freie Universität Berlin, Königsweg 67, D-14163, Berlin, Germany

**Keywords:** Cattle, Trypanosomosis risk, Trypanocide resistance, Mali

## Abstract

**Background:**

Tsetse fly-transmitted African animal trypanosomosis causes annual losses that run into billions of dollars. The disease is assumed to cause hunger and poverty in most sub-Saharan countries since it represents a serious impediment to sustainable livestock production. Both a cross-sectional and a longitudinal study were carried out from November to December 2007 to evaluate trypanosomosis risk and susceptibility of trypanosomes to trypanocidal drug treatment in village cattle populations in south-east Mali.

**Methods:**

Eight purposively selected villages participated in the study. In each village, eight traps deployed along drainage lines over 24hour duration were used to catch tsetse. One hundred systematically selected cattle in the study villages were examined for trypanosomes. All trypanosome-positive cattle were randomly allocated into two treatment groups: a group treated with 0.5 mg/kg bw. isometamidium chloride (ISMM) and a group treated with 3.5 mg/kg bw. diminazene aceturate (DIM). The cattle were monitored for trypanosomes at day 14 and 28 post-treatment.

**Results:**

Of the 796 cattle examined, 125 (15.7%) were trypanosome-positive. Village trypanosome prevalences ranged between 11% and 19%. There were no significant (p > 0.05) differences in the village trypanosome prevalences. *Trypanosoma congolense* was the dominant trypanosome species accounting for 73% (91/125) of the infections and *T. vivax* the remainder. Twenty (31.7%) of the 63 cattle on 0.5 mg/kg bw. ISMM treatment were still positive14 days post-treatment. Of the 43 aparasitaemic cattle monitored to day 28, 25.6% (11) became parasitaemic, resulting in a cumulative failure rate of 49.2% (31/63). *Trypanosoma congolense* accounted for 77.4% (24/31) of failed ISMM treatments. The 62 cattle treated with 3.5 mg/kg bw. DIM resulted in 30.6% (19/62) failed treatments. Although 42.2% (19/45) of *T. congolense* positive cattle did not respond to DIM treatment, all *T. vivax* positive cattle responded positively to DIM treatment.

**Conclusions:**

The overreliance on trypanocides in the control of trypanosomosis will ultimately lead to multiple drug-resistant trypanosome populations as detected in villages in south-east Mali rendering the use of drugs doubtful. Effective alternative methods for trypanosomosis control ought to substitute chemotherapy to ensure sustainable cattle production in these villages. Since there is no single strategy for containing trypanocidal drug resistance, promotion of an integrated approach combining proven trypanosomosis control approaches in high trypanosomosis risk areas is most desirous. The best-bet strategy this study recommended for areas with multiple drug resistance included area-wide community tsetse control, control of co-infections to exploit self-cure against resistant trypanosome populations and the rational use of trypanocidal drugs which should be urgently promoted at all levels as a way of containing or reversing resistance.

## Background

In sub-Saharan Africa (SSA), tsetse fly-transmitted African animal trypanosomosis (AAT) is estimated to cause annual losses that run into billions of dollars [1]. AAT is indeed considered one of the root causes of hunger and poverty in most SSA countries where it represents a serious impediment to sustainable agricultural rural development (http://www.africa-union.org/Structure_of_the Commission/dep-Pattec.htm). About 80% of land in SSA is tilled by hand due to the high risk of AAT that threatens the survival and use of draught animals. In south-east Mali for instance, AAT outbreaks in the 1980s killed trypano-susceptible zebu cattle introduced in the 1960s for draught power leaving most households without oxen for traction [2].

Chemotherapy is and will continue to be preferred in the control of trypanosomosis. Vector control requires donor support for sustainability while trypanotolerant cattle are a minority in the cotton zone of West Africa [3]. Only a small group of chemo-prophylactic and chemo-therapeutic compounds are currently in use with new anti-trypanosomal compounds or anti-infection vaccine unlikely to be available in the near future [4]. Unfortunately, the rapid development of resistance to the available drugs presents a serious threat that will potentially render them ineffective in trypanosomosis control [4]. The dependence of smallholder farming on trypanocides, the serious negative effects of drug resistance and the fact that resistance will worsen and spread if nothing is done, warrant action to prevent and contain the problem of drug resistance.

In the cotton zone of West Africa, reports of trypanocidal drug resistance were first made in south-west Burkina Faso in the 1980s [5,6]. This was followed by multiple drug resistance reports in the same country [7]. Comprehensive epidemiological surveys across the cotton zone of West Africa have revealed high prevalences of trypanosomes and trypanocidal drug resistance in a wider area stretching from south-west Burkina Faso across southern Mali into north-east Guinea [8-10]. Unlike in Burkina Faso where drug resistance situation has clearly been established through a number of studies [5-7,10], information on this subject in south-east Sikasso is still scanty. This paper describes results of a study undertaken to confirm trypanocidal drug resistance in south-east Sikasso to set the scene for testing of integrated best-bet strategies for containing and or reversing resistance.

## Methods

### Study area description

The study was carried out in Sikasso District, south-east Mali. Sikasso lies on longitude 11° 19’ N, latitude 5° 40’ W at an altitude of 410 m above sea level. The district is situated on the eastern side of Sikasso Region, which is bordered to the east by Burkina Faso, Guinea Conakry to the west, to the south by Côte d’Ivoire and by Koulikoro and Segou Regions to the north and north-east. The region has a sub-humid climate typical of the pre-guinean savannah zone with 3 seasons: cold dry season from November to January, hot dry season from February to May and rainy season from June to October [8]. Sikasso receives ~1000-1200 mm of rainfall annually, peaking in July and August which makes it one of the areas in Mali with the highest agricultural potential [11]. Mixed crop-livestock farming is the main source of livelihoods. Maize, sorghum, millet and rice are the main subsistence crops while cotton and groundnuts are the main cash crops. Other crops grown include root crops (yams, sweet and Irish potatoes), legumes, fruit trees, and small-scale horticultural crops, particularly vegetables.

Sikasso landscape is dominated by a series of river valleys featuring riparian vegetation types that are still largely undisturbed providing a favourable habitat for the *palpalis* (Nemorhina) group of tsetse flies. Savannah flies formerly present in Sikasso and in other areas of the cotton zone of West Africa are believed to have since disappeared [12] following the destruction of their habitat through opening up of more land for settlement and food crop cultivation.

### Cattle breeds and production system

Trypano-susceptible zebu cattle account for about 80% of cattle in the study area, with the remainder composed of crosses (*métis*) of zebu with the indigenous trypano-tolerant cattle breeds, the *Méré* and N’Dama [13]. Cattle are kept for traction, savings as “living banks” and performing social obligations (dowry and rituals). Cattle also provide milk and meat [14]. Cattle were extensively grazed on communal pastures with minimal and occasional supplements (discarded mango fruit, maize and millet bran). During the cropping season (rainy season), cattle are grazed away from farms but allowed to graze on crop residues after harvesting with their dung directly fertilizing fields in readiness for the next cropping season.They are watered in rivers or at community bore holes when seasonal rivers dry out. Draught oxen are grazed and watered close to the homesteads throughout the year. Herd owners keep animals together in an enclosure at night, releasing the herd to graze during the day. Fulani pastoralists from the north bring transhumant herds to Sikasso in the south during the dry season, creating seasonal or permanent complementary and competitive relationships between the settled agro-pastoralist communities and the immigrant pastoralist groups, sometimes leading to conflicts [14].

### Study villages

**Figure 1:**
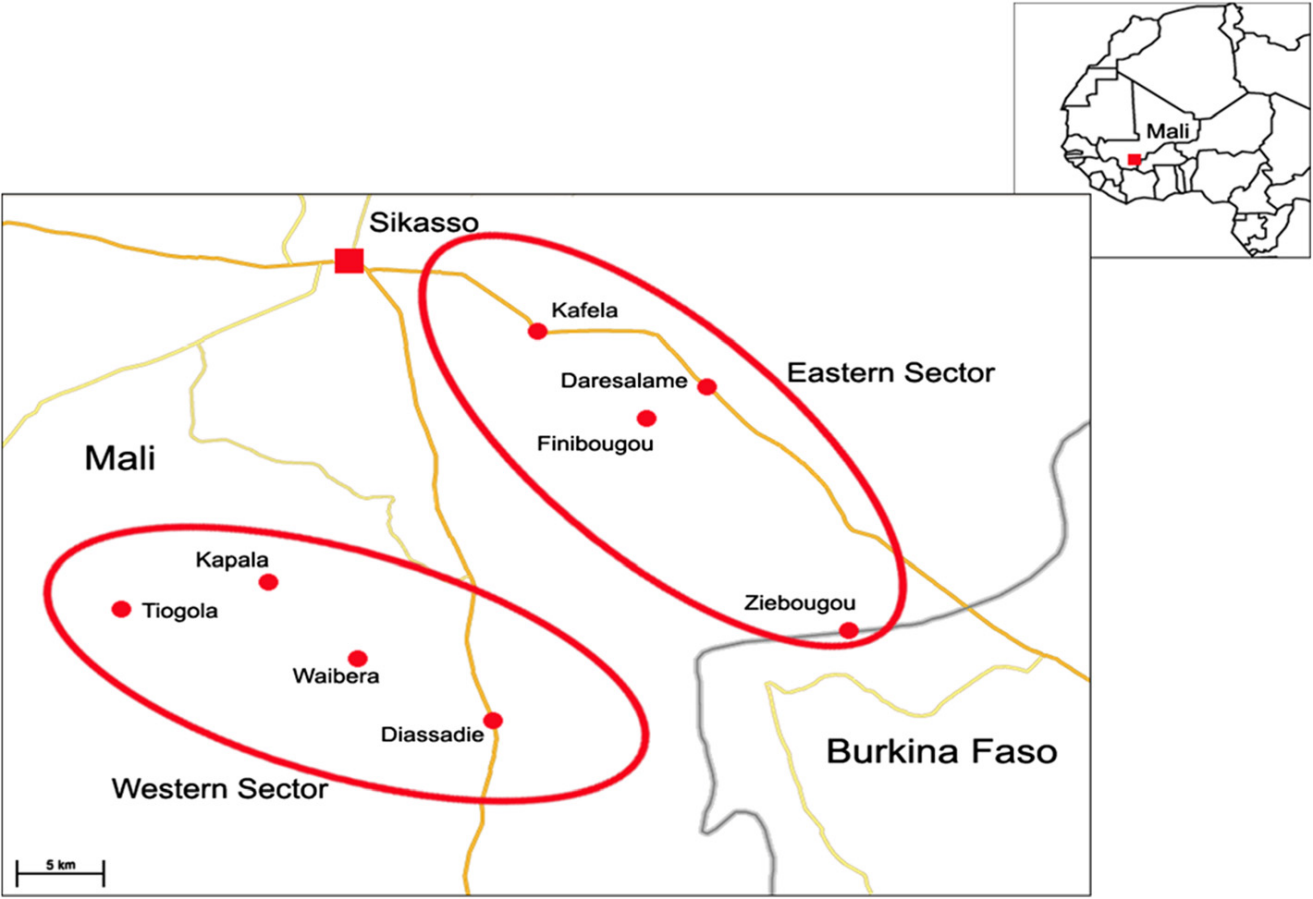
Study map showing the study sites (circled) and study villages (dotted red), south-east Mali.

Eight villages participated in this study. The villages were purposively pre-selected from a list of 25 villages due to high prevalence of trypanosomes in cattle (> 10%) and suspected trypanocidal drug resistance [9,10]. Half of the selected villages were located along the road from Sikasso to Burkina Faso, termed the eastern sector, and the other half along the road from Sikasso to Côte d’Ivoire, constituting the western sector (Figure 1). The study villages had comparable agricultural production systems, vegetation type and river drainage system.

### Study design

Cross-sectional and longitudinal surveys were conducted in November-December 2007. The cross-sectional survey helped to estimate tsetse density, trypanosome prevalence in village cattle and trypanocide use practices while the longitudinal survey established trypanocidal drug resistance levels.

### Tsetse survey

A cross-sectional entomological survey was conducted in November 2007 to establish tsetse fly densities. Eight unbaited and untreated bi-conical traps [15] per village were deployed at an interval of about 100–200 m in the forest galleries along the rivers. The traps were emptied 24 hours later and flies counted, identified, and separated into their respective species and sex.

### Cattle sampling and trypanosome prevalence

A systematic sampling approach was used to select study cattle [16]. Briefly, the first animal in every village herd was randomly sampled and thereafter every second animal until the 100 sample size was reached. This sampling was adopted because the cattle population per village was approximately 200. Only cattle aged at least 12 months were examined for trypanosomes from each of the 8 study villages as per recommendation [17]. A jugular vein blood sample was extracted from every cattle into vacutainer tubes containing di-sodium salt of ethylene diamine tetra-acetate (EDTA) and evaluated for packed cell volume (PCV) using the Hawksley microhaematocrit reader (Hawksley, Lancing, United Kingdom) and for trypanosomes using the dark-ground phase microscopy [18].

### Drug sensitivity study

Trypanosome-positive cattle from each village were randomly allocated into a group treated with isometamidium chloride (ISMM) (Trypamidium®, Merial, France) or diminazene aceturate (DIM) (Veriben®, Ceva Animal Health Inc., France). The cattle were ear-tagged and their weights estimated using a weighing band and conversion tables developed for Mali’s local zebu cattle [19]. The two drugs were reconstituted in distilled water as per the manufacturers’ recommendation; the ISMM group was treated with 0.5 mg/kg body weight (bw.) of 2% solution and the DIM group treated with 3.5 mg/kg bw. of 7% solution through a deep intramuscular (i.m.) injection by an animal health assistant under supervision of a team of veterinarians. The treatment day was considered as day 0. Treated cattle were monitored for trypanosomes and PCV on days 14 and 28 post-treatment. Trypanosome-positive cattle in both groups at days 14 and 28 were retreated with 7 mg/kg bw. DIM. This block treatment study lasted 4 weeks (28^th^ November to 28^th^ December 2007).

### Data analysis

Entomological data were summarized by tsetse species and presented as the number of flies per trap per day (FTD). Differences in fly densities were determined using the Kruskal Wallis test. Trypanosome-positive cattle were classified by trypanosome species and Pearson chi square (χ^2^) used to test differences in trypanosome prevalence between villages. Treatment response was given by drug and monitoring date. ISMM treatment response was considered at days 14 and 28 post-treatment whereas DIM treatment response was considered only at day 14 post treatment due to its short prophylactic activity [20]. Fisher exact test compared treatment failure between villages while Student’s t-test was used to determine differences in PCV values. Persisting trypanosomes in the ISMM or DIM treated cattle were indicative of resistance to the respective drug. Data were analysed using SPSS version 18. The online program OpenEpi (http:/http://www.openepi.com) was used to calculate confidence intervals.

## Results

### Tsetse survey

Two riverine tsetse species, *Glossina palpalis gambiensis* and *G. tachinoides*, were encountered in the study area. A total of 580 tsetse flies were caught (Table 1). *Glossina p. gambiensis* was the dominant tsetse species with FTD of 6.8 in the eastern sector and FTD of 4.4 in the western sector. For *G. tachinoides, *a mean FTD of 4.0 was recorded in the eastern sector and a mean FTD of 2.9 in the western sector. Although fly catches varied across villages, the eastern sector registered slightly higher mean catches than the western sector.

**Table 1:** ***Glossina* caught in 8 traps/village in south-east Mali (November to December 2007)**

**Villages**	***G. p. gambiensis***^***1***^		***G. tachinoides***^***2***^		**Total fly catch**	
	**No. flies**	**Flies/trap/day (FTD)**	**No. flies**	**Flies/trap/day (FTD)**	**No. flies**	**Flies/trap/day (FTD**)
**Eastern sector**						
Kafela	27	3.4	26	3.3	53	6.6
Finibougou	94	11.8	64	8.0	158	19.8
Daresalame	63	7.9	37	4.6	100	12.5
Ziébougou	35	4.4	2	0.3	37	4.6
**Subtotal flies/mean FTD**	**219**	**6.8**^**a**^	**129**	**4.0**^**b**^	**348**	**10.9**
**Western sector**						
Diassadié	43	5.4	22	2.8	65	8.1
Waibera	72	9.0	47	5.9	119	14.9
Kapala	15	1.9	1	0.1	16	2.0
Tiogola	10	1.3	22	2.8	32	4.0
**Subtotal flies/mean FTD**	**140**	**4.4**^**a**^	**92**	**2.9**^**b**^	**232**	**7.3**
**Total flies/mean FTD**	**359**	**5.6**	**221**	**3.6**	**580**	**9.1**

^1^ *G. p. gambiensis* = *Glossina palpalis gambiensis*.

^2^ *G. tachinoides* = *Glossina tachinoides*.

Same letter superscripts denote along column of comparison denote no statistical difference (Kruskal Wallis test, p > 0.05) in FTD for the two sectors.

### Trypanosome prevalence and PCV levels

Of the 796 cattle that were examined in the 8 villages, 125 (15.7%) were trypanosome-positive (Table 2). Although the trypanosome prevalence was slightly higher in the western sector villages than in the eastern sector villages, there was no significant difference between the villages (p > 0.05). *Trypanosoma congolense* was the dominant trypanosome species and accounted for 73% (91/125) of all the trypanosome infections. No *T. brucei* or mixed infections were detected.

**Table 2:** Trypanosome prevalence and mean PCV (%) in cattle in south-east Mali (November to December 2007)

**Villages**	**Trypanosome positive cattle**			**No. of cattle examined**	**Prevalence (%)**	**95% CI**^**3**^	**Mean PCV% (95% CI)**
	***T.c.***^***1***^	***T. v.***^***2***^	**Total**				
**Eastern sector**							
Kafela	10	4	14	100	14.0	8.2-21.9	28.4 (27.2-29.5)
Finibougou	14	2	16	100	16.0	9.8-24.2	26.4 (25.5-27.4)
Daresalame	12	2	14	96	14.6	8.5-22.7	29.0 (28.1-30.0)
Ziébougou	5	6	11	100	11.0	5.9-18.3	25.4 (24.6-26.2)
**Subtotal and mean PCV (%)**	**41**	**14**	**55**	**396**	**13.9**^**a**^	**10.7-17.6**	**27.3 (26.8-27.8)**
**Western sector**							
Diassadié	17	2	19	100	19.0	12.2-27.6	26.2 (25.3-27.2)
Waibera	14	4	18	100	18.0	11.4-26.5	23.9 (23.0-24.8)
Kapala	10	4	14	100	14.0	8.2-21.9	25.8 (24.8-26.8)
Tiogola	9	10	19	100	19.0	12.2-27.6	26.2 (25.3-27.2)
**Subtotal and mean PCV (%)**	**50**	**20**	**70**	**400**	**17.5**^**a**^	**14.0-21.5**	**25.5 (25.0-26.0)**
**Total and mean PCV (%)**	**91**	**34**	**125**	**796**	**15.7**	**13.4-18.3**	**26.4 (26.1-26.8)**

^1^ T.c. = *Trypanosoma congolense*.

^2^ T.v. = *T. vivax*.

^3^95% CI = 95% confidence interval.

PCV% = Percent packed cell volumes.

Same letter superscript denote no statistical significance (χ² test, p > 0.05) differences in trypanosome prevalence along columns of comparison.

Mean PCV in the studied cattle was 26.4% (95% CI: 26.1-26.8%) with those from the eastern sector having a significantly (p < 0.05) higher mean PCV of 27.3% compared to mean PCV of 25.5% for the cattle in the western sector (Table 2). Trypanosome-positive cattle had significantly (p < 0.05) lower PCV (23.2%) compared to trypanosome-negative cattle (27.0%). Among the trypanosome-positive cattle, those infected with *T. congolense* had a lower PCV (22.7%) compared to the cattle infected with *vivax* (24.7%). This difference was not significantly (p > 0.05) different.

### ISMM treatment response

At day 14 post-treatment, trypanosome susceptibility to 0.5 mg/kg bw. ISMM varied across the study villages (Table 3). Overall, of the 63 trypanosome-positive cattle treated at this dose, 31.7% (20/63) still had persistent infections. Despite the high ISMM treatment failure in the eastern sector villages, the difference between the sectors was not significant (p > 0.05). Among the ISMM treated cattle, *T. congolense* accounted for about 90% (18/20) of the failed treatments. *Trypanosoma vivax* positive cattle in the eastern sector that received ISMM treatment were all cleared of these trypanosomes as opposed to cattle in the western sector where a few *T. vivax* still persisted following treatment with ISMM.

**Table 3:** Cattle with failed isometamidium chloride treatment over total treated in south-east Mali (November to December 2007)

**Villages**	**Treatment response 14 days post-treatment**			**Treatment response 28 days post-treatment**^**1**^			**Cumulative treatment response**^**2**^
	***T.c.***^***3***^	***T. v.***^***4***^	**Total**	***T.c.***^***3***^	***T. v.***^***4***^	**Total**	
**Eastern sector**							
Kafela	1/4 (25.0)	0/3 (0)	1/7 (14.3) ^5^	1/3 (33.3)	2/3 (66.7)	3/6 (50.0)	4/7 (57.1)
Finibougou	4/7 (57.1)	0/1 (0)	4/8 (50.0)	1/3 (33.3)	0/1 (0.0)	1/4 (25.0)	5/8 (62.5)
Daresalame	4/6 (66.7)	0/1 (0)	4/7 (57.1)	0/2 (0.0)	1/1 (100.0)	1/3 (33.3)	5/7 (71.4)
Ziebougou	3/3 (100.0)	0/3 (0)	3/6 (50.0)	-	0/3 (0.0)	0/3 (0.0)	3/6 (50.0)
**Subtotal**	**12/20 (60.0)**	**0/8 (0)**	**12/28 (42.9)**^**a**^	**2/8 (25.0)**	**3/8 (37.5)**	**5/16 (31.3)**^** c**^	**17/28 (60.7)**^**d**^
**Western sector**							
Diassadie	3/9 (33.3)	0/1 (0.0)	3/10 (30.0)	2/6 (33.3)	0/1 (0.0)	2/7 (28.6)	5/10 (50.0)
Waibera	0/7 (0.0)	0/2 (0.0)	0/9 (0.0)	1/7 (14.3)	1/2 (50.0)	2/9 (22.2)	2/9 (22.2)
Kapala	2/5 (40.0)	1/2 (50.0)	3/7 (42.9)	0/3 (0.0)	0/1 (0.0)	0/4 (0.0)	3/7 (42.9)
Tiogola	1/5 (20.0)	1/4 (25.0)	2/9 (22.2)	1/4 (25.0)	1/3 (33.3)	2/7 (28.6)	4/9 (44.4)
**Subtotal**	**6/26 (23.1)**^**b**^	**2/9 (22.2)**	**8/35 (22.9)**^**b**^	**4/20 (20.0)**	**2/7 (28.6)**	**6/27 (22.2)**^**c**^	**14/35 (40.0)**^**d**^
**Total**	**18/46 (39.1)**	**2/17 (11.8)**	**20/63 (31.7)**	**6/28 (21.4)**	**5/15 (33.3)**	**11/43 (25.6)**	**31/63 (49.2)**

^1^Cattle that were aparasitaemic at day 14 and monitored for trypanosomes at day 28;

^2^Cumulative treatment response = trypanosome positive cattle at day 14 post-treatment added to those positive 28 days post-treatment;

^3^ *T.c.= Trypanosoma congolense*.

^4^ *T.v.* = *Trypanosoma vivax*.

^5^Number of cattle with failed treatment over number of cattle treated (percent treatment failure rate).

Different letter superscripts along the column of comparison are significantly (p < 0.05) different.

Of the 43 cattle aparasitaemic at day 14 post-treatment, 25.6% (11/43) were trypanosome-positive on day 28. *Trypanosoma congolense* accounted for 54.5% (6/11) of the failed treatments (Table 3). The cumulative treatment failure rate (summing days 14 and 28 failed ISMM treatments) was 49.2% (31/63). *Trypanosoma congolense* accounted for 77.4% (24/31) of all ISMM treatment failures. The eastern sector had a higher treatment failure (60.7%) compared to the western sector (40%). This was however not significantly different (p > 0.05).

### DIM treatment response

Trypanosome response to DIM treatment varied across the study villages (Table 4). Of the 62 trypanosome-positive cattle treated with 3.5 mg/kg bw. DIM at day 0, 30.6% (19/62) had persistent trypanosomes 14 days later. Eastern sector villages had the highest DIM treatment failures compared to the western sector villages. *Trypanosoma congolense* accounted for all failed DIM treatments in both sectors. Comparatively, all *T. vivax* strains were apparently sensitive to 3.5 mg/kg DIM. Re-treatment of *T. congolense* positive cattle which had failed DIM with double DIM dose (7 mg/kg DIM) still resulted in a treatment failure rate of 26.3% (5/19). The eastern sector still had a slightly higher failure rate than the western sector.

**Table 4:** Cattle with failed diminazene aceturate treatment over total treated in south-east Mali (November to December 2007)

**Villages**	**Treatment response 14 days post-treatment with 3.5 mg/kg bw. DIM**			**Response at day 14 post retreatment with 7.0 mg/kg bw. DIM**^**1**^
	***T. congolense***	***T. vivax***	**Total**	***T. congolense***
**Eastern sector**				
Kafela	5/6 (83.3)^2^	0/1	5/7 (71.4)	2/5 (40.0)
Finibougou	2/7 (28.6)	0/1	2/8 (25.0)	0/2 (0.0)
Daresalame	2/6 (33.3)	0/1	2/7 (14.3)	1/2 (50.0)
Ziebougou	1/2 (50.0)	0/3	1/5 (20.0)	0/1 (0.0)
**Subtotal**	**10/21 (47.6)**	**0/6 (0)**	**10/27 (37.0)**	**3/10 (30.0)**
**Western sector**				
Diassadie	2/8 (25.0)	0/1	2/9 (22.2)	0/2 (0.0)
Waibera	2/7 (28.6)	0/2	2/9 (22.2)	1/2 (50.0)
Kapala	3/5 (60.0)	0/2	3/7 (42.9)	1/3 (33.3)
Tiogola	2/4 (50.0)	0/6	2/10 (20.0)	0/2 (0.0)
**Subtotal**	**9/24 (37.5)**	**0/11 (0)**	**9/35 (25.7)**	**2/9 (22.2)**
**Total**	**19/45 (42.2)**	**0/17 (0)**	**19/62 (30.6)**	**5/19 (26.3)**

^1^Retreatment with 7.0 mg/kg bw. DIM was done on cattle that had relapsed trypanosomes 14 days after treatment with 3.5 mg/kg bw. DIM.

^2^Number of cattle parasitaemic 14 days after treatment over number of cattle treated (treatment failure rate %).

### Response of PCV level to treatment

Treatment of the trypanosome-positive cattle with DIM or ISMM significantly (p < 0.001) increased the PCV% from 23.2 ± 5.5% at day 0 to 27.3 ± 6.3% at day 14 and eventually to 29.5 ± 6.0 at day 28.

## Discussion

*Glossina palpalis gambiensis* and *G. tachinoides* were the only tsetse species detected in the study area*,* with the former dominating. Savannah tsetse species were not detected during this survey. They are believed to have disappeared from the area following destruction of their habitat [12] hence tsetse trapping by this study was limited to the drainage lines where *palpalis* species are found. The presence of riverine tsetse species has important implications for tsetse control planning since emphasis must be placed on riparian habitats.

A variability in tsetse catches was observed across the study villages during this survey. The FTD for *G. p. gambiensis (5.6)* was considerably lower than the FTD 15–20 reported for the same species across the border in the Samorogouan pastoral area of Burkina Faso [21]during surveys in 1994.

This study confirmed that trypanosomosis is an important disease in Sikasso. Variability in trypanosome prevalence across study villages was similar to what was observed in the Kénédougou Province of Burkina Faso [8]. Trypanosome prevalence measured in this study is consistent with those for the same area [9,11]. Also consistent with the two earlier studies, *Trypanosoma congolense* dominated *T. vivax*. The same pattern had been observed in neighbouring Kénédougou Province in Burkina Faso [7,8]. Since *T. congolense* is the most pathogenic trypanosome of cattle its dominance is likely to increase trypanocidal drug use and in the process accelerate the development of resistance against trypanocidal drugs [22].

One of the undeniable pathogenic effects of trypanosome infections in cattle is the occurrence of anaemia, indicated by low PCV [23-25]. The present study found the expected relationship with trypanosome-positive cattle having significantly lower PCV on average than negative ones. The results also suggested that *Trypanosoma congolense*-positive cattle had lower mean PCV than *T. vivax*-positive cattle, though not statistically confirmed. This agrees with what was observed in Kénédougou Province in Burkina Faso, Mandiana in Guinea and south-east and south west Mali [14] and in a pastoral area of south-western Burkina Faso [25].

Monitoring effectiveness of treatments given to cattle under field conditions and drug sensitivity tests in mice and ruminants under laboratory conditions are used to characterize susceptibility of trypanosomes to trypanocidal drugs [17]. Drug sensitivity studies are generally expensive, time-consuming and labour intensive. Doing sensitivity studies in mice has an additional disadvantage that *T. vivax* and some *T. congolense* do not readily grow in mice. *In-vitro* assays are suitable for characterizing *T. brucei* although they are time-consuming [26]*.* The drug incubation *Glossina* infectivity test (DIGIT) is a sensitive method [27] although its use is dependent on the availability of laboratory-reared tsetse flies. Some genetic markers have been developed [28,29] but their use is very limited due to lack of appropriately equipped laboratories and skilled personnel in SSA.

In the present study, an abbreviated 28-day field protocol based on treatment of naturally infected cattle [9] was preferred over the longer follow-up protocol [17]. Although there was risk of underestimating the resistance as detection was only limited to parasitological methods [30], the abbreviated protocol was still preferred over other resistance characterization methods because of certain advantages. Firstly, this protocol uses cattle of known trypanosome infection status and takes only one month to generate results at a reasonably low cost. Secondly, the abbreviated protocol makes it possible to simultaneously estimate resistance of both DIM and ISMM. Lastly, the drop-out rate of participating herd owners/study animals where the abbreviated protocol is applied is lower than for the long follow-up protocol. To increase the precision of resistance estimates, the survey was timed to coincide with the period of highest trypanosomosis risk (end of the rainy season) to ensure a large population of naturally trypanosome-infected cattle.

Within the cattle cohort that received ISMM treatment, persistent trypanosomes were detected 14 days post-treatment across the study villages indicative of resistance against this drug. *Trypanosoma congolense* accounted for about 90% of the ISMM resistant trypanosomes. The few cattle with *T. vivax* failed ISMM treatments were mostly detected at day 28 post-treatment which seemed to suggest that at high plasma ISMM concentrations, *T. vivax* are effectively suppressed, only flourishing as ISMM plasma concentration wanes.

Re-treatment of ISMM treatment failures with 7 mg/kg DIM led to the apparent clearance of all persistent *T. vivax* infections. Since residual levels of ISMM are still present in animals, this is likely to influence the treatment outcome of the second drug, hence caution is suggested when interpreting such parasitological outcomes [8].

There was high variability in the DIM treatment failures across the study villages. Indeed, molecular analysis using PCR-RFLP technique of filter paper blood samples collected during the drug sensitivity test confirmed presence of DIM resistant *T. congolense* populations in the study area [31]. A slightly higher percentage of treatment failure (36.8%) after DIM treatment was observed in the Kénéndougou Province of Burkina Faso [8]. The sensitivity of *T. vivax* to 3.5 mg/kg DIM treatment reported by this study is consistent with earlier findings of other studies [7,8]. Re-treatment of the cattle positive for persistent *T. congolense* with a double dose (7 mg/kg) of DIM did not completely clear trypanosomes in 26.3% of the re-treated cattle. Doubling the dose of DIM only slightly improved therapeutic efficacy on *T. congolense* strains resistant to 3.5 mg/kg DIM [32]*.* In other studies, higher doses like 17.5 mg/kg DIM [7] and 14 mg/kg DIM [21] still failed to clear the *T. congolense* resistant to 3.5 mg/kg DIM.

## Conclusions

The results of this study confirmed the presence of apparent multiple drug-resistant *T. congolense* and isometamidium resistant *T. vivax* populations in village cattle of south-east Mali. This scenario renders the use of sanative drug pairs in the control of trypanosomosis untenable. In areas with confirmed resistance, an integration of trypanosomosis control should be encouraged as opposed to over-reliance on chemotherapy as the sole strategy. Application of integrated strategies aimed at lowering the risk of transmission (vector control) and supplementary health enhancing packages for bolstering immunological responses in trypanosome-infected cattle would be of great benefit in terms of complete elimination of the resistant trypanosome populations from an area.

## Competing interests

The authors declare that they have no competing interests.

## Authors’ contributions

EOM carried out the field surveys, assembled data, and drafted the manuscript. HSV, EMC, ZB, BD and YS carried out the field surveys and reviewed the manuscript. OD, TFR, BB and PHC participated in study coordination, design and revised the manuscript. KHZ reviewed the manuscript. All authors read and approved the final manuscript.
